# Posterior reversible encephalopathy syndrome with lumbar drainage and surgery: coincidence or correlation? A case report

**DOI:** 10.1186/s12883-019-1438-8

**Published:** 2019-08-30

**Authors:** Brent G. Oxford, Nicolas K. Khattar, Shawn W. Adams, Alexandra S. Schaber, Brian J. Williams

**Affiliations:** Department of Neurological Surgery, University of Louisville School of Medicine, 220 Abraham Flexner Way, 15th Floor, Louisville, KY 40202 USA

**Keywords:** Posterior reversible encephalopathy syndrome, Lumbar drain, Intracranial hypotension, Sellar mass

## Abstract

**Background:**

Posterior reversible encephalopathy syndrome (PRES) is a rare neurological disorder usually associated with specific medical conditions that cause a disturbance of the CNS homeostasis. It has seldom been reported to be a consequence of an iatrogenic intervention causing intracranial hypotension.

**Case presentation:**

We report the case of an individual 69-year-old male presenting with headache and blurred vision following cerebrospinal fluid (CSF) leak from resection of a sellar mass. The patient developed the condition following removal of the lumbar drain post-operatively. Magnetic Resonance Imaging showed bilateral occipital, parieto-occipital, and cerebellar T2 FLAIR hyper-intensities, suggesting a radiological diagnosis of posterior reversible encephalopathy syndrome (PRES). The patient’s symptoms started to improve shortly afterwards and had completely resolved at 3 months follow-up.

**Conclusions:**

The absence of severe hypertension and presence of an intraoperative CSF leak requiring placement of the lumbar drain suggests that decreased CSF volume and associated reactive hyperemia could have a role in the pathophysiology of the disease.

## Background

Posterior reversible encephalopathy syndrome (PRES) originally described by Hinchey includes neurological abnormalities and associated characteristic findings on neuroimaging [1]. PRES is most commonly associated with eclampsia although it may occur in infections, autoimmune disease, cancer chemotherapy, transplantation, and hypertension [1–3]. Clinical symptoms range from headache, altered mental status, vision changes including cortical blindness, paresis, seizures, and nausea with severe cases leading to coma and death [1–3]. Computerized Tomography (CT) or Magnetic Resonance Imaging (MRI) typically shows a reversible focal symmetric hemispheric edema involving the watershed zones, often in the parietal and occipital lobes [2, 4–6]. In this report, we present the case of a patient with PRES associated with intracranial hypotension after removal of a lumbar drain.

### Case presentation

Our patient is a 69-year-old Caucasian male with known hypertension, hyperlipidemia, gout and sialolithiasis presenting for endoscopic endonasal resection of a recurrent intra/suprasellar mass with cavernous sinus extension with pathological specimen consistent with Rathke’s cleft cyst. Cerebrospinal fluid (CSF) leak occurred intra-operatively requiring placement of a lumbar drain at a rate of 10 cc/h. CSF pressure was not elevated during the initial placement. Drainage continued intermittently at the same rate for 48 h. The drain was then clamped for 24 h prior to removal on post-operative day three without adverse event. The patient did not complain of any headaches following the surgical intervention but developed a severe positional headache associated with nausea, vomiting, and significantly blurred vision. The headaches were worsened upon sitting upright and partially relieved in the recumbent position. The ophthalmological examination demonstrated decreased visual acuity at 20/30 bilaterally. The optic disc retained spontaneous venous pulsations, suggestive of low or normal intracranial pressure.

**Fig. 1:**
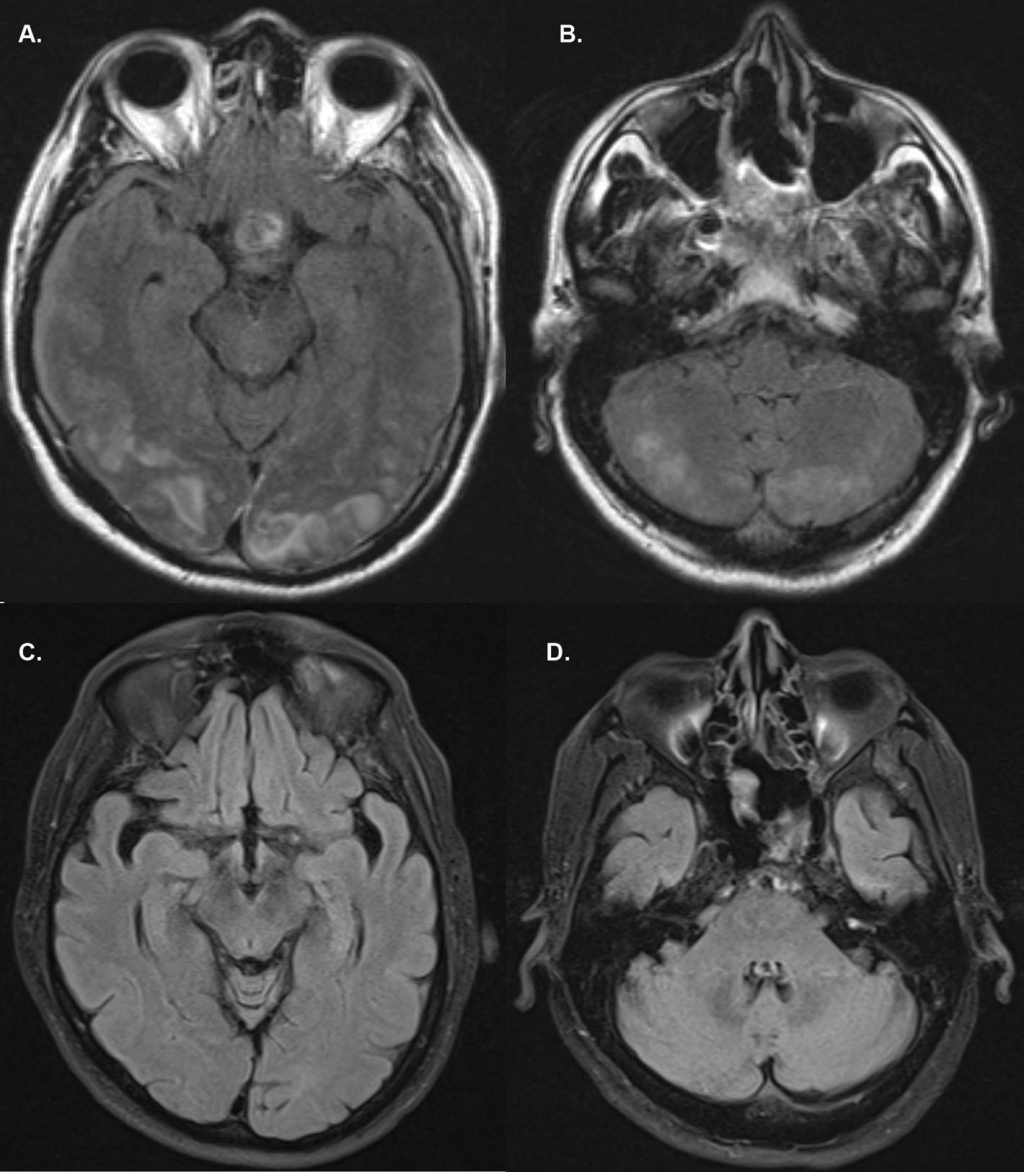
Axial brain MRI reveals patchy areas of increased T2 FLAIR hyper-intensity in the bilateral occipital lobes (**a**) and posterior cerebellum (**b**). Follow-up MRI at 6-months follow-up show complete resolution of the areas of T2 FLAIR hyper-intensity in the bilateral occipital lobes (**c**) and posterior cerebellum (**d**)

CT of the head revealed subcortical hypodensities at the posterior tip of the occipital lobes bilaterally. MRI demonstrated patchy areas of T2 and T2 FLAIR hyper-intensity in the bilateral posterior occipital lobes as well as the bilateral parieto-occipital junction and posterior cerebellum (Fig. 1).

**Fig. 2:**
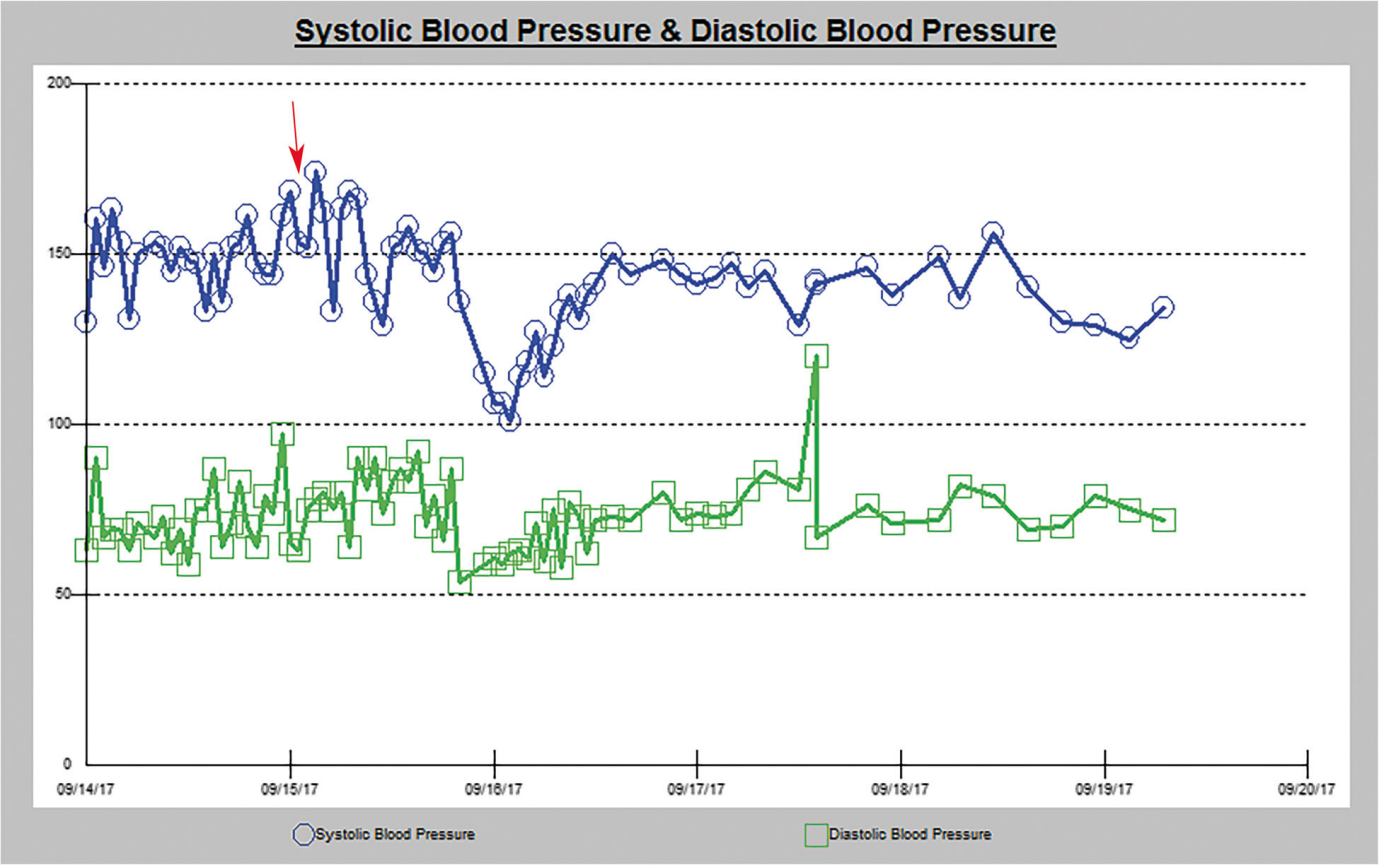
Systolic and diastolic blood pressure variation during the entire hospitalization. The red arrow depicts the time of the lumbar drain removal

The patient’s systolic blood pressure is charted in Fig. 2. The systolic blood pressure ranged between 101 and 174 mmHg and the diastolic blood pressure ranged between 54 and 120 mmHg. The red arrow (Fig. 2) represents a blood pressure of 152/80 mmHg and corresponds to the exact timing of the lumbar drain removal. The patient had a history of hypertension and was maintained on his home oral anti-hypertensive medications. The acute variations in his systolic blood pressure were being treated with intravenous anti-hypertensives. No CSF leak was noted post-operatively and supportive therapy was continued. The patient’s symptoms improved one day later with his visual acuity normalizing on ophthalmological follow-up examination. He was discharged on post-operative day eight with continued improvement of his symptoms at six months follow-up. MRI follow-up at six months showed complete resolution of the T2 lesions (Fig. 1).

## Discussion and conclusion

Posterior reversible encephalopathy syndrome is a rare disorder, and its mechanism remains controversial. It has been previously described occurring following lumbar CSF drainage [7–10]. One of the prevailing theories suggests that severe hypertension can precipitate cerebral auto-regulatory failure leading to hyperperfusion, endothelial injury and subsequently fluid extravasation [11, 12]. Hypertension may be associated with reflexive vasoconstriction leading to ischemic injury damaging the blood brain barrier and allowing subsequent extravasation [13]. PRES is known to occur in normotensive patients and such a tight association with hypertension is less likely, given that the latter is only present in 70–80% of cases [4]. In our case, the patient’s blood pressure was elevated at 152/80 immediately prior to the removal of the lumbar drain. There could be a potential association of CSF drainage following clamping of the lumbar drain for 24 h and restoration of normal intrathecal CSF volume, with the instantaneous occurrence of hypertension that led to PRES in that situation. Such CSF drainage would be caused by the presence of a lumbar drain-induced durotomy in the lumbar cistern that would leak additional fluid prior to sealing with compression.

Another potential mechanism of action of PRES involves endothelial dysfunction leading to an increase in membrane permeability and vasogenic edema as seen on imaging [14, 15]. Even though the mechanism of endothelial dysfunction is mostly unknown, manipulation of areas of cardiovascular regulation such as the hypothalamus (in the setting of a Rathke’s cleft cyst resection) could trigger the endothelial dysfunction [16]. This initial insult can trigger an immune response with the release of various cytokines leading to disruption of tight junctions causing vasogenic edema [14]. This inflammatory cascade leading to disruption of vascular endothelium potentially offers an explanation linking the various conditions that have been documented to cause PRES independently of blood pressure abnormalities [2, 14, 15].

Recently, intracranial hypotension has also been associated with PRES [17]. Demonstrating the occurrence of intracranial hypotension is challenging given the absence of ICP measurement in our patient. However, an intraoperative CSF leak and the continued lumbar CSF drainage post-operatively decreased the volume of CSF, thereby decreasing CSF pressure. This phenomenon may lead to increased perfusion pressure and passive edema [18]. Increased perfusion pressure could potentially lead to over-distension of cerebral veins and arterioles causing hydrostatic extravasation of fluid [17]. Reduced CSF volume leading to increased perfusion has been reported following repeated lumbar punctures or lumbar drainage thereby leading to the development of PRES [9, 10]. Pressure-volume relationships have been extensively studied and have fit most closely with a parabolic regression model [19–21]. The application of the Monroe-Kellie doctrine implies that a decrease in CSF volume is met with a compensation in the occupation of the space in the cranium. Since the brain cannot significantly expand, this leads to a resultant hyperemia. This has been correlated with post-lumbar puncture headaches and the effectiveness of caffeine in treating them [22–25]. Inflammation has been extensively shown to disrupt the blood-brain barrier [26]. The immune background possibly associated with PRES could lead to the increased hydrostatic extravasation when paired with hyperemia secondary to CSF volume depletion [27, 28]. In our patient, CSF volume was decreased due to the intraoperative CSF leak and the lumbar drainage. The patient had mounted a background inflammatory response to the surgical intervention three days prior to the inciting event. Following the removal of the drain, the patient possibly experienced an acute additional decrease in CSF volume and pressure. In the setting of increased inflammation post-operatively, that could have possibly incited the extravasation of fluid and the development of symptoms. The absence of continued CSF leak and the restoration of the normal distribution of the intracranial volume following removal could explain the rapid resolution of the symptoms. The occurrence of PRES is very rare in post-operative patients, which indicates that other underlying pathways may be involved. The limitation of this study is the fact that this phenomenon occurred in a single patient only.

PRES is a potential consideration in post-operative patients with CSF leak with sudden neurological changes, specifically deficits in vision or seizures. The mechanism of PRES remains controversial and further investigations should be conducted whenever it does occur to advance our understanding of its etiology.

## Data Availability

The datasets used and/or analyzed during the current study are available from the corresponding author on reasonable request.

## Abbreviations

CNS: Central nervous system
CPP: Cerebral perfusion pressure
CSF: Cerebrospinal fluid
CT: Computed Tomography
FLAIR: Fluid-attenuated Inversion recovery
ICP: Intracranial pressure
MAP: Mean arterial pressure
MRI: Magnetic Resonance Imaging
PRES: Posterior Reversible Encephalopathy Syndrome
